# Osseous Manifestations of Primary Hyperparathyroidism: Imaging Findings

**DOI:** 10.1155/2020/3146535

**Published:** 2020-02-21

**Authors:** Jackson Bennett, James W. Suliburk, Fanny E. Morón

**Affiliations:** ^1^School of Medicine, Baylor College of Medicine, Houston, TX, USA; ^2^Department of Surgery, Baylor College of Medicine, Houston, TX, USA; ^3^Department of Radiology, Baylor College of Medicine, Houston, TX, USA

## Abstract

Primary hyperparathyroidism is a systemic endocrine disease that has significant effects on bone remodeling through the action of parathyroid hormone on the musculoskeletal system. These findings are important as they can aid in distinguishing primary hyperparathyroidism from other forms of metabolic bone diseases and inform physicians regarding disease severity and complications. This pictorial essay compiles bone-imaging features with the aim of improving the diagnosis of skeletal involvement of primary hyperthyroidism.

## 1. Introduction

Hyperparathyroidism (HPT) is an endocrine disorder defined by a state of inappropriately increased levels of parathyroid hormone (PTH) activity from one or more parathyroid glands [1]. Primary hyperparathyroidism (PHPT) is a main disease subtype with classical and normocalcemic variants. Hypercalcemia is evident in patients with classical PHPT, while the normocalcemic variant demonstrates normal total and ionized calcium levels after correcting for albumin [2–4]. Classical PHPT is most commonly asymptomatic and due to autonomous secretion of parathyroid hormone from a benign parathyroid adenoma in 80% of patients with lack of feedback inhibition of calcium [1, 4, 5]. Multigland disease (double adenoma, triple adenoma, and spontaneous four-gland hyperplasia), parathyroid carcinoma, and syndromic forms of PHPT typically comprise the remaining 20% of cases [5–7]. The normocalcemic variant is characterized by secondary elevations in PTH without an exact known etiology. This variant may represent a subclinical, asymptomatic early stage of PHPT, and it has the potential to progress to hypercalcemic status in 20% of cases and cause the target end organ damage seen in the symptomatic classical variant in some individuals [2, 4]. Other HPT disease subtypes include secondary and tertiary disease, which are primarily seen in patients with chronic renal disease and posttransplant patients [7].

Bone is a major target organ of PTH, and inappropriately elevated PTH levels in PHPT can lead to changes in the appearances of bones on a variety of diagnostic imaging evaluations. Metabolic bone disease is an established clinical manifestation of PHPT as PTH is a major regulator of osteoclast activity and bone remodeling [8]. In addition, the biomechanical properties of bone as seen in PHPT's variants, including fracture risk and protective bone treatment, are an area of ongoing scientific interest [3]. However, the clinical and physical symptoms as well as imaging findings that have been historically taught are considered relatively rare today in clinical presentation and context. This is thought to be due to earlier diagnosis and regular evaluations of calcium. From an epidemiological disease perspective of PHPT, the prevalence in the United States of the classical hypercalcemia form has been estimated to be 0.86% overall with a certain degree of variability [9, 10]. Many cases of Western PHPT are now initially identified in otherwise asymptomatic patients through routine biochemical screening in up to 2% of patients over the age of 55 [1, 4, 11], while more severe, overt radiographical bone disease is rarer with potential to carry complications [1, 6, 12]. Widened prevalence estimates also exist for other disease phenotypes such as the normocalcemic form of PHPT (0.4–11%). Additionally, the modern incidence and prevalence estimates of PHPT have increased regionally in the USA, Europe, and China, which is thought to be attributed to a worldwide increase in routine biochemical disease screening. This is contrasted with developing countries in which PHPT presents with higher serum calcium levels and more symptomatic disease compared to the asymptomatic hypercalcemia seen in the USA, Europe, and China [9, 10].

There is an overlap regarding the bone imaging findings of primary and secondary HPT. While secondary HPT is not specifically discussed in depth here, it has a unique osteosclerotic effect on the axial skeleton that produces a classic “rugger-jersey spine” [13]. In this pictorial essay, we mainly focus on the skeletal findings of PHPT bone disease as seen on multiple imaging modalities. We present an organized discussion on subperiosteal bone resorption-acroosteolysis, subchondral bone resorption, brown tumors of the body, salt-and-pepper skull, and osteopenia associated with PHPT. The overall aim of this pictorial essay is to review this wide range of musculoskeletal imaging findings associated with PHPT in addition to common differential conditions to aid in diagnosing and enhancing our knowledge of this enigmatic disease.

### 1.1. Subperiosteal Bone Resorption

Subperiosteal bone resorption corresponds to destruction of the bone underneath the cortical periosteum of long bones; it is due to increased bone turnover. The mechanism of heightened levels of bone turnover is due to the unregulated effects of parathyroid hormone on bone calcium homeostasis as seen in PHPT [5]. Elevated PTH levels lead to upregulation of nuclear factor-*κβ* ligand (RANKL), which interacts with its respective RANK receptor on osteoclast progenitor cells, leading to resorption via the indirect growth of bone-remodeling osteoclasts [8, 14]. These findings in the phalanges are defining, pathognomonic musculoskeletal imaging features of primary HPT [15].

**Figure 1:**
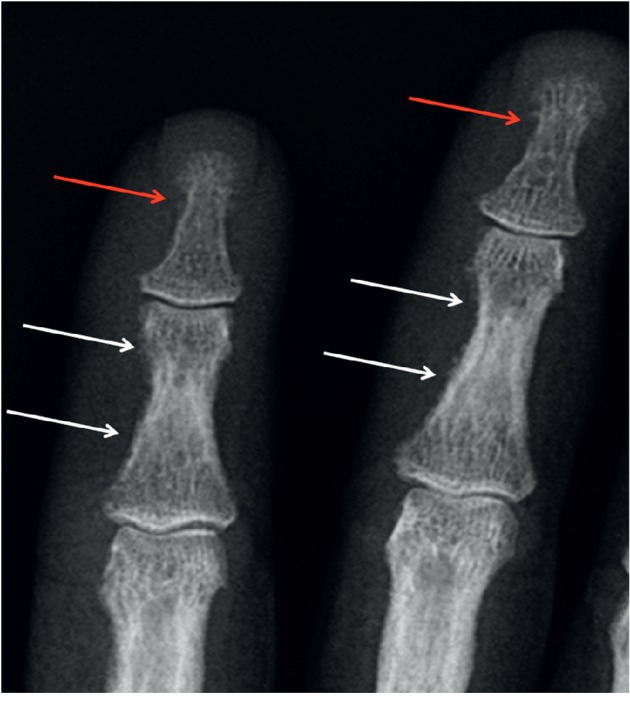
Subperiosteal resorption of the radial aspect of the middle phalanges of the second and third fingers (white arrows); feathery appearance and early tufts resorption-acroosteolysis (red arrows).

**Figure 2:**
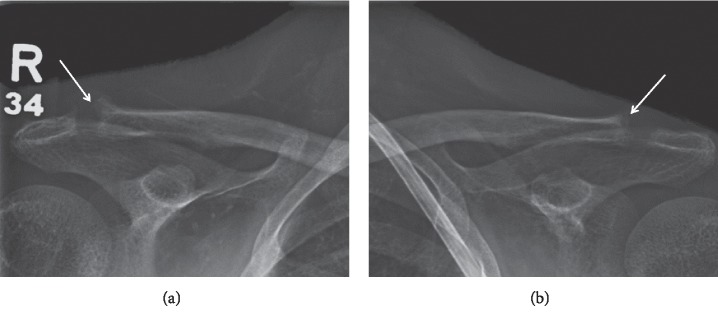
Clavicular subchondral resorption (arrows), widening of the articular space and irregular-feathery articular surface bilaterally.

**Figure 3:**
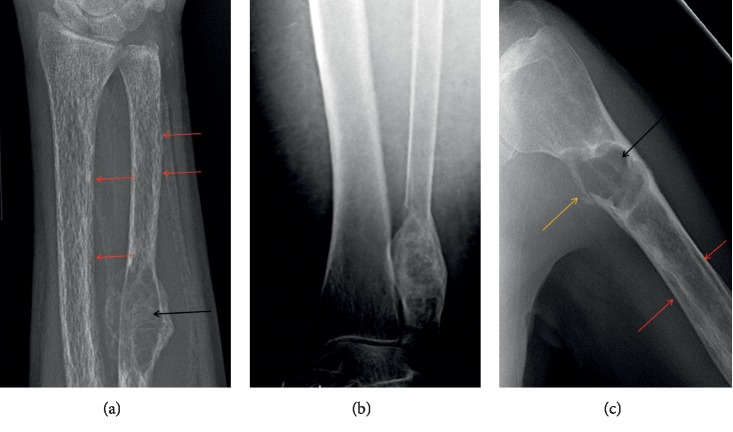
Additional cases of brown tumors (black arrows) in the ulna, fibula, and humerus. Prominent forearm demineralization with subperiosteal (red arrows), intracortical, and trabecular resorption in the forearm bones and humerus with resultant appearance of coarse internal trabeculation. Pathologic fracture at the humerus brown tumor (yellow arrow) and intracortical resorption with cigar/oval-shaped or tunnel-shaped radiolucency in the cortex (red arrows).

**Figure 4:**
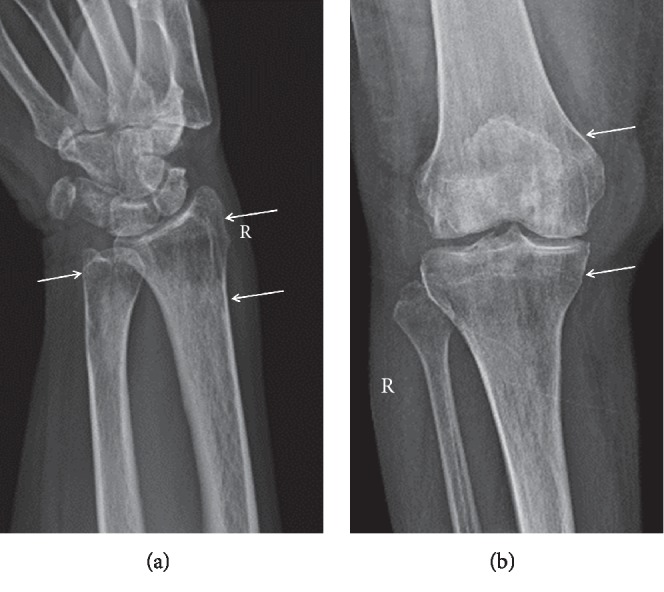
Osteoporosis. Demineralization with predominance of the distal radius and ulna and around the knee, with cortical thinning due to subperiosteal resorption (arrows).

The osseous changes most commonly occur at the proximal and middle phalanges located on the radial margins of the second and third fingers, and they are best viewed on radiographs [13]. On radiographs, subperiosteal bone resorption appears as lace-like subperiosteal/intracortical irregular margin of phalangeal cortical bone [5, 12] with thinned and feathery cortical bone (Figure 1). While elevated serum calcium levels in the setting of high PTH activity ensure a probable diagnosis of PHPT, a majority of patients who have skeletal changes associated with PHPT (up to 95% of patients) are best assessed radiographically at the hand, highlighting this location as a very specific osseous finding of the disease [5, 16]. Thus, when clinical findings suggest PHPT, radiography is the preferred imaging study, specifically at the hand, to look for subperiosteal resorption if osseous involvement is a concern [16]. In addition to the hand, subperiosteal resorption, cortical thinning, and acroosteolysis can also be seen in the long bones (Figures 2–4), the lamina dura of teeth and spine [5, 17]. Intracortical resorption can also occur and it appears on radiographs as cigar/oval-shaped or tunnel-shaped radiolucency within the cortex (Figure 3). In combination, subperiosteal bone resorption and the brown tumors of osteitis fibrosis cystica (OFC) contribute to 2% of symptomatic HPT manifestations on bone [18]. The radiographic differential diagnosis for subperiosteal arthritis, like changes in the hand, includes rheumatoid arthritis.

Acroosteolysis is a type of subperiosteal bone resorption pattern seen in PHPT and renal osteodystrophy that is located at the distal phalangeal tufts. Diffuse or “band-like” radiolucent patterns of resorption may occur, characteristically at the midshaft and distal phalanx of a single or multiple digits. Key radiological findings to note entail tuft destruction (Figure 1), soft tissue tapering and shortening at distal phalanx, a lucent line crossing the middle phalanx, calcifications, and arthritic changes. Other etiologies of this osseous finding include scleroderma, vascular, infectious, inflammatory, thermal injury, and traumatic and congenital causes of distal phalangeal shortening. Since acroosteolysis of the phalanges can occur in various other conditions, it is important to correlate these findings with other associated clinical and imaging features of HPT [5, 15].

### 1.2. Subchondral Resorption around Specific Joints

**Figure 5:**
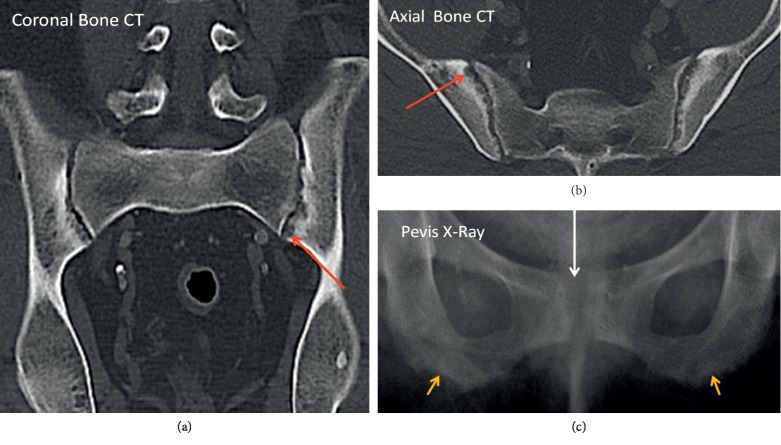
Subchondral resorption of the sacroiliac joints. Coronal and axial CT images show areas of subchondral lucency with irregular articular margin (red arrows), apparent widening of the joint space and surrounding hyperdense sclerosis. Pelvis X-ray symphysis pubis subchondral resorption (white arrow) with widening and also subligamentous resorption of the ischial tuberosities (yellow arrows). (a) Coronal bone CT. (b) Axial bone CT. (c) Pelvis X-ray.

Subchondral resorption is an osseous abnormality with trabecular destruction underneath cartilage surfaces of articular spaces; there is also fibrous replacement and new bone formation as well as juxta-articular erosions [13, 17]. The pathophysiology occurs due to PTH-mediated osteoclastic resorption of bone and surrounding cartilage [13]. Osteoclastic bone remodeling is upregulated by the resorption of PHPT, exerting its effect on joints with tight articulation, which are vulnerable to excessive shear stresses. Histologically, these changes may resemble woven bone in addition to fibrous tissue underneath cartilage [17]. Commonly affected joints comprise the acromioclavicular (Figure 2) and sternoclavicular joints, phalanges, pubic symphysis, and sacroiliac joints (Figure 5) [15].

On radiographs, subchondral resorption leads to widening of the articular space (as the result of collapsed resorted bone) and irregular appearance of the articular surfaces with indistinct articular margin [5, 15] (Figures 2 and 5). The erosions seen at the sacroiliac joint also tend to occur on the iliac sides [5, 15, 17] (Figure 5). In addition, at the acromioclavicular joint, bilateral erosions tend to affect the clavicular side more than the acromion (Figure 2), whereas the sternum and clavicle are equally affected at the sternoclavicular joint. Reactive sclerosis may additionally be present (Figure 5). The osseous changes at the hand, sacroiliac joints, and pubic symphysis may mimic the findings of other differential diagnoses such as the inflammatory arthridites and seronegative spondyloarthropathies [17].

### 1.3. Focal Lytic Lesions

A lytic lesion corresponds to localized bone loss creating an area of lucency in bone. The lytic lesions associated with HPT are named brown tumors, osteolytic aggregate of cyst-like entities seen in long-standing hyperparathyroidism termed OFC. The histology is similar to that of giant cell tumors, appearing as multinucleated giant cells; the bone marrow is replaced by reparative, richly vascularized connective tissue secondary to rapidly increased osteoclastic resorptive activity. Hemosiderin accumulation from hemorrhages associated with this vascularized tissue accounts for their characteristic brown color [18]. Brown tumors are associated with PHPT in up to 3% of patients [18, 19]. The incidence of brown tumors is now also increasingly common in secondary HPT (1.5–1.7% of patients) due to the prevalence of renal disease, dialysis, and this condition in comparison [5, 19, 20]. Brown tumors can be single or multiple and may be located in any site. The most common sites of brown tumors associated with PHPT include the pelvis, mandibles, ribs, long bones, and hands in addition to the vertebrae [5, 18, 21].

**Figure 6:**
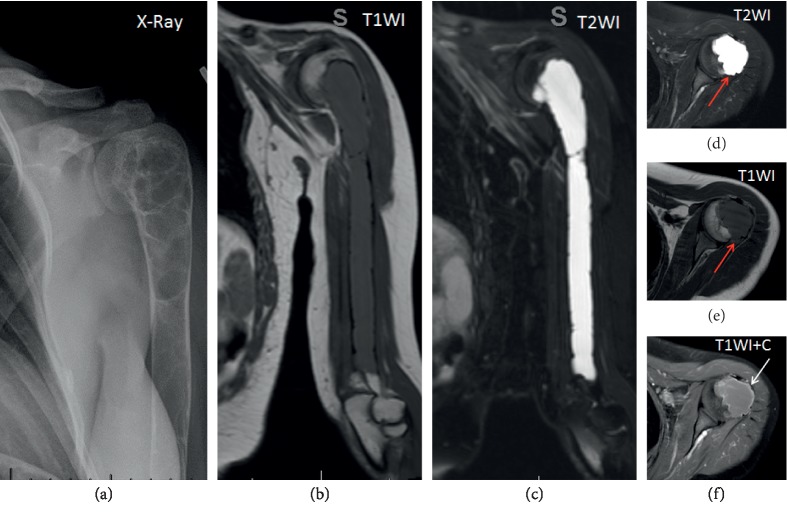
Left humerus brown tumor. X-Ray shows a large, well defined, multiloculated soap-bubbly lucent lesion. MRI T1W1 the lesion is similar to the muscle, on coronal and axial T2WI the lesion is hyperintense (very bright), axial T1WI without contrast the lesion is hypoinetense with prominent thinning of the cortical bone and minimal extension beyond the cortex (red arrows), axial T1WI + C with contrast and fat saturation shows diffuse and peripheral enhancement (white arrow).

**Figure 7:**
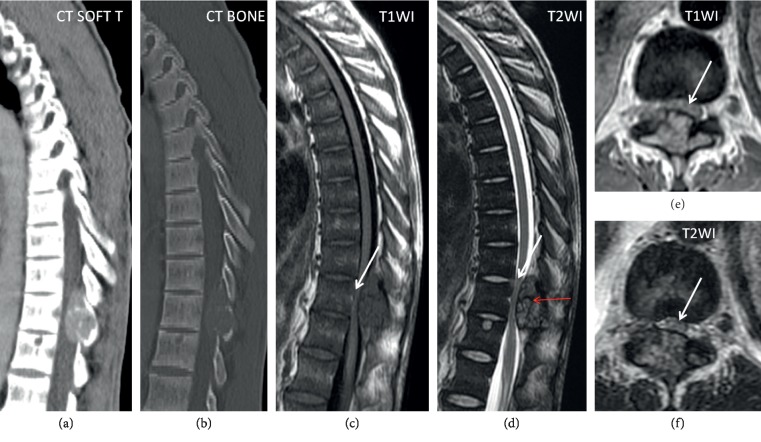
Thoracic spine brown tumor. Sagittal CT, sagittal T1WI/T2WI, and axial T1WI/T2WI MRI sequences show an expansible, well-circumscribed lesion in the posterior elements extending within the spinal canal, with severe spinal canal stenosis and compressed spinal cord (white arrow). Areas of low signal on T2W1 from hemosiderin are indicated by the red arrow.

On radiographs, brown tumors of PHPT appear as expansile, solitary, or multifocal well-defined soap-bubbly appearing lytic lesions with cortical thinning (without associated periotic reaction [20, 22]). Additionally, these lesions are characterized as having a narrow zone of transition into the normal bone but no reactive changes in the adjacent bone and well-defined sclerotic margins. There is adjacent cortical thinning but usually not frank destruction or breakthrough (Figure 6). Computed tomography (CT) further localizes the lesion in 3 planes within the bone marrow and proximity to the cortical bone or articular surfaces; CT better depicts cortical thinning and breakthrough as well as associated pathologic fractures. On CT, the lesion is expansile, lytic, and well circumscribed; the density varies depending on the relative proportion of its components. The lesions usually contain a combination of solid, cystic, and hemorrhagic components. The cystic component has lower attenuation (hypodense or darker density); the solid component has higher attenuation (hyperdense or whiter density) (Figure 7), and if there has been a recent bleed, the blood will be even denser, and adjacent soft tissue component may be present [5, 20]. Nuclear medicine bone scans can demonstrate intense brown tumor focal uptake [15]. Magnetic resonance imaging (MRI) is performed to further evaluate the extension of these lesions into adjacent compartments and associated complications such as pathologic fractures or spinal canal invasion and encroachment of the spinal cord. Traditional sequences include T1- and T2-weighted images (T1WI and T2WI, respectively) and fat-saturated sequences. The signal intensity of the lesion is inhomogeneous on MRI. Degraded blood products can cause high signal on T1WI and scattered areas of lower T2WI signal throughout the lesion (Figure 7). Like on CT, the lesion heterogeneity depends on the relative proportion of the lesion components. Solid component is similar to muscle on T1WI and T2WI; the cystic component is hyperintense on T2WI (Figure 6) and may also present with fluid-fluid levels on T2WI. Usually, the administration of contrast is not warranted; if administered, it may present inhomogeneous and peripheral enhancement [22] (Figure 6). The imaging differential diagnosis of brown tumors includes other lucent and lytic bone lesions, such as true giant cell tumors, multiple myeloma, fibrous dysplasia, and metastases [15]. Brown tumors can be clinically differentiated from these conditions based off of serum calcium, serum electrophoresis, and correlated with an isotope bone scan or other radiographic findings suggestive of PHPT [15, 23].

Spinal brown tumors represent a rare manifestation of OFC when compared to established locations such as the ribs, pelvis, mandible, and long bones [24]. The thoracic spine may be involved in up to 57% of spine cases [24]. Multilevel involvement of the spine affecting various vertebral bodies and posterior spinal elements has also been reported [24, 25]. Clinically, brown tumor spinal involvement is particularly significant because while it may present asymptomatically, it carries the extra potential complication of lesional growth and extension into the spinal canal. This can result in canal stenosis and compression of the spinal cord with frank neurological deficits at presentation requiring emergent decompressive surgery and parathyroidectomy [21, 24, 25]. The imaging findings of the spinal brown tumors are nonspecific and the same as elsewhere in the body (Figure 7). MRI is the preferred diagnostic imaging modality in evaluating spinal tumor location and extension. Solitary spinal lesions have a differential diagnosis of metastases, giant cell tumor, aneurysmal bone cyst, and giant cell reparative granuloma, which can be clinically and radiographically correlated with other findings of PHPT [26]. In addition, the typical CT and MRI findings in patients with a lytic lesion and known PHPT are highly suggestive of a brown tumor diagnosis as opposed to other conditions in the differential such as multiple myeloma [24, 27]. It is generally accepted that PHPT patients with overt symptomatic disease should undergo parathyroidectomy if they are reasonable surgical candidates [28]. From a treatment standpoint, parathyroidectomy has been shown to result in complete brown tumor regression [29, 30]. While parathyroidectomy is considered first line for treatment of OFC, surgical management of osseous lesions is debated and may be considered in certain patients [31]. These situations occur in the setting of misdiagnosis, delays in treatment, or lack of biochemical screening, which are more commonly seen in PHPT patient populations of developing countries [31]. Examples of lesions that may require surgery include those that fail to regress or have extensive brown tumor involvement of surrounding structures, for which local bone surgical intervention may be warranted [16, 29, 31]. Other cases entail large, aggressive brown tumors associated with severe pain, delayed treatment, pathological fractures leading to disability, or recurrence after parathyroidectomy [31].

### 1.4. Salt-and-Pepper Skull

**Figure 8:**
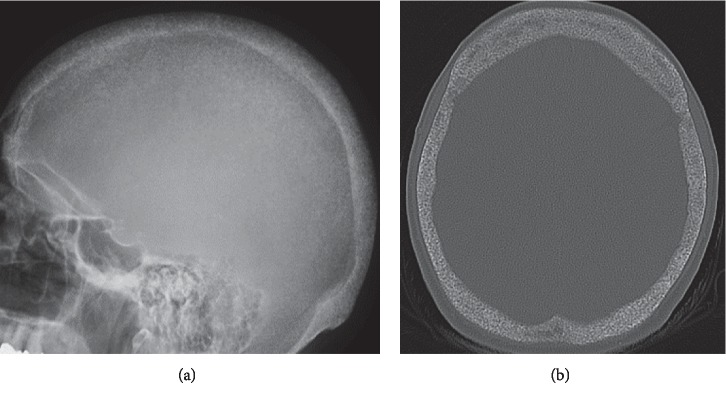
“Salt-and-pepper-skull.” Lateral skull X-ray and axial bone windows CT with salt-and-pepper appearance from trabecular bone resorption depicted as fine areas of lucency mixed with sclerotic radiopaque-denser-dot-like foci.

Salt-and-pepper skull refers to diffuse, lytic foci interspersed between regular bone in the calvarium giving a granular skull appearance that occurs as a result of HPT [32]. In PHPT, the pathogenesis involves trabecular bone resorption that leads to decreased differentiation of the diploic space bone marrow and the inner and outer tables of the calvarium [5]. On imaging, bone demineralization and deossification create punctate, lucent foci and a generalized, ground-glass image associated with smudgy trabeculae and focal areas of patchy sclerosis described in combination as the “salt-and-pepper” skull appearance [5, 33] (Figure 8). These findings can be readily visualized on radiographs and CT. Altogether, these findings may be the first imaging change seen in patients presenting with HPT or as a component of OFC [33, 34]. The expanded differential diagnosis of skull demineralization includes osteoporosis associated with aging and less commonly anemias such as sickle cell and thalassemia, HPT, metastatic bone disease, multiple myeloma, and the lytic phase of Paget disease [35]. In differentiating the calvarium salt-and-pepper skull of PHPT from other lesions of the calvarium, further confirmation with patient characteristics, clinical history, laboratory analysis, and imaging in combination is essential [32].

### 1.5. Osteopenia

Osteopenia is defined by the World Health Organization as a state of decreased bone density with a densitometry T-score of –1 to –2.5 standard deviations less than that of a young healthy reference population [5, 36, 37]. In PHPT, mechanistically, there is resorption of secondary trabeculae (which corresponds to interlinking non-weight-bearing trabeculae) and accentuation of primary trabeculae (weight-bearing trabeculae) and overall decreased bone density due to the increased osteoclastic activity and vascularized fibrous tissue seen in PHPT. Generalized and asymmetric osteopenia is the most common skeletal finding in modern-day PHPT [7, 16, 31]. Recent studies cite the prevalence of osteoporosis in PHPT ranging from 39 to 62.9% [7]. The radiographic finding of decreased bone density (more lucent bones) requires 30–50% bone loss to be detected by human perception [14]. Other radiographic findings include accentuation vertical striation of primary trabeculae (coarse internal trabeculation (Figure 3)), which corresponds to weight-bearing vertically oriented ticked trabeculae within the lucent background. Cortical thinning appears as sharply demarcated distinction between cortex and medullary cavity due to accentuation of the cortical lining (Figures 3 and 4). It is important to note that decreased bone mineral density can also occur in senile, postmenopausal, and secondary causes of osteoporosis [38]. The osteopenia of PHPT preferentially affects the peripheral skeleton rather than the axial skeleton, which differs from the pattern seen in senile osteoporosis and postmenopausal women osteoporosis [38]. While PHPT is now commonly discovered as an asymptomatic disease, some studies have shown that bone mass loss in these patients is more than would be anticipated despite the lack of pathognomonic radiograph findings [12].

Currently, methods for assessing the severity of bone disease typically include radiographs, dual-energy and peripheral X-ray absorptiometry (DXA), and quantitative ultrasonography [39]. Additional technologies include high-resolution CT and a Trabecular Bone Score (TBS) to gauge osteoporotic fracture risk factors [40, 41]. Historically, the bone alterations seen in PHPT are described as having a predilection for cortical rather than trabecular osseous compartments. The mechanism of this finding is thought to be multifactorial, including an increased susceptibility of cortical bone to excess PTH over time and the nature of bone turnover in these regions [42, 43]. While trabecular bone has been previously viewed as relatively preserved in PHPT, emerging imaging techniques such as high-resolution CT suggest a significant correlation in conjunction with TBS in demonstrating both cortical and trabecular bone microalterations at sites such as the distal radius and tibia [44]. TBS is employed as an indirect measure of trabecular bone microarchitecture and is currently being researched as a useful complement to DXA measurements of bone [12, 45]. In a study of postmenopausal women with PHPT, TBS demonstrated trabecular network alterations in the presence of PHPT that are not readily directly detectable by DXA, notably in the microarchitectural analysis of the lumbar spine of asymptomatic patients with milder forms of disease [12, 44]. When correlated with transiliac bone biopsy in a cohort of male and female patients, TBS has also been shown to demonstrate value as a surrogate technique for analyzing trabecular bone microarchitecture alterations [46, 47].

Clinically, the bone demineralization seen in PHPT is important to recognize early on as osteopenia has potential to progress into frank osteoporosis and predispose patients to pathological fragility fractures in the spine and forearm [5, 14]. Of special consideration are fractures of the vertebrae, which are composed of roughly 70% cancellous bone and can be a presenting, clinically silent symptom in patients with mild, untreated PHPT despite a preference of PTH for cortical bone [7, 48, 49]. A population-based cohort study by Khosla et al. of patients diagnosed with PHPT showed that these patients exhibited increased vertebral, distal forearm, rib, pelvic, and overall risk of fracture with marginal increase in the risk of femoral fractures. This study supports the theory that excess levels of PTH have a significant effect on cancellous bone in addition to cortical bone [48]. The ability of trabecular imaging modalities to detect more extensive skeletal deterioration than previously demonstrated by conventional densitometric imaging is important, as it has led to discussions regarding expanding evaluation and criteria for definitive parathyroidectomy in patients with PHPT [50].

In patients with decreased bone mineral density associated with PHPT (Figure 4), parathyroidectomy helps to restore bone health [12]. In a subset of patients with PHPT and low lumbar spine bone density, up to a 20% remineralization has been seen 4 years post-op from parathyroidectomy [14, 51]. At time frames of 6 months post-op, several studies have shown BMD improvements at the lumbar spine and hips [43, 52]. Additionally, PHPT's demineralizing effect on cortical bone at the femoral neck and distal one-third radius has been found to be at least partially reversible by parathyroidectomy 10–15 years post-op [14, 43]. There is evidence to suggest a sustained, gradual increase in femoral neck BMD that can occur after parathyroidectomy [46]. Overall, parathyroidectomy is seen as the curative and definitive treatment of this condition with significant improvements to BMD and reductions in nephrolithiasis, although it is unclear as to whether there are marked improvements in bone strength and fragility fracture risk [7, 14, 28].

## 2. Conclusion

The bone imaging manifestations of PHPT are diverse with skeletal findings on radiographs that are very characteristic of the disease. The common symptomatic osseous findings include subperiosteal and subchondral joint resorption, acroosteolysis, the salt-and-pepper skull, the brown tumors of OFC, and osteopenia. Knowledge of the classic imaging patterns and differential diagnosis in combination with the clinical picture of PHPT is invaluable for informing clinicians in preventing misdiagnosis, providing earlier intervention, and counseling patients on what is a curable disease with resultant improvement in imaging pathology.

## Abbreviations

HPT: Hyperparathyroidism
PTH: Parathyroid hormone
PHPT: Primary hyperparathyroidism
RANKL: Nuclear factor-*κβ* ligand
RANK: Nuclear factor-*κβ*
OFC: Osteitis fibrosa cystica
CT: Computed tomography
MRI: Magnetic resonance imaging
WI: Weighted images
DXA: Dual-energy X-ray absorptiometry
TBS: Trabecular bone score
25(OH)D: 25-Hydroxy-vitamin D
1,25-(OH)2D: 1,25-Dihydroxy-vitamin D.

## References

[1] Bilezikian J. P., Cusano N. E., Khan A. A., Liu J.-M., Marcocci C., Bandeira F. (2016). Primary hyperparathyroidism nature reviews. *Disease Primers*.

[2] Bilezikian J. P., Brandi M. L., Eastell R. (2014). Guidelines for the management of asymptomatic primary hyperparathyroidism: summary statement from the fourth international workshop. *The Journal of Clinical Endocrinology & Metabolism*.

[3] Bollerslev J., Schalin-Jäntti C., Rejnmark L. (2019). Unmet therapeutic, educational and scientific needs in parathyroid disorders: consensus statement from the first European society of endocrinology workshop (PARAT). *European Journal of Endocrinology*.

[4] Zini M., Attanasio R., Cesareo R. (2012). AME position statement: primary hyperparathyroidism in clinical practice. *Journal of Endocrinological Investigation*.

[5] Chang C. Y., Rosenthal D. I., Mitchell D. M., Handa A., Kattapuram S. V., Huang A. J. (2016). Imaging findings of metabolic bone disease. *RadioGraphics*.

[6] Khan A., Bilezikian J. (2000). Primary hyperparathyroidism: pathophysiology and impact on bone. *CMAJ: Canadian Medical Association Journal*.

[7] Walker M. D., Silverberg S. J. (2018). Primary hyperparathyroidism. *Nature Reviews Endocrinology*.

[8] Rolighed L., Rejnmark L., Christiansen P. (2014). Bone involvement in primary hyperparathyroidism and changes after parathyroidectomy. *European Endocrinology*.

[9] Press D. M., Siperstein A. E., Berber E. (2013). The prevalence of undiagnosed and unrecognized primary hyperparathyroidism: a population-based analysis from the electronic medical record. *Surgery*.

[10] Silva B. C., Cusano N. E., Bilezikian J. P. (2018). Primary hyperparathyroidism. *Best Practice & Research Clinical Endocrinology & Metabolism*.

[11] Pradeep P. V., Jayashree B., Mishra A., Mishra S. K. (2011). Systematic review of primary hyperparathyroidism in India: the past, present, and the future trends. *International Journal of Endocrinology*.

[12] Bandeira F., Cusano N. E., Silva B. C. (2014). Bone disease in primary hyperparathyroidism. *Arquivos Brasileiros de Endocrinologia & Metabologia*.

[13] Murphey M. D., Sartoris D. J., Quale J. L., Pathria M. N., Martin N. L. (1993). Musculoskeletal manifestations of chronic renal insufficiency. *Radiographics*.

[14] Marcocci C., Cianferotti L., Cetani F. (2012). Bone disease in primary hyperparathyrodism. *Therapeutic Advances in Musculoskeletal Disease*.

[15] Manaster B. J. (2010). Diagnostic imaging: non-traumatic disease.

[16] (2017). Imaging in primary hyperparathyroidism: practice essentials, radiography, magnetic resonance imaging.

[17] Resnick D., Niwayama G. (1976). Subchondral resorption of bone in renal osteodystrophy. *Radiology*.

[18] Jouan A., Zabraniecki L., Vincent V., Poix E., Fournié B. (2008). An unusual presentation of primary hyperparathyroidism: severe hypercalcemia and multiple brown tumors. *Joint Bone Spine*.

[19] Takeshita T., Tanaka H., Harasawa A., Kaminaga T., Imamura T., Furui S. (2004). Brown tumor of the sphenoid sinus in a patient with secondary hyperparathyroidism: CT and MR imaging findings. *Radiation Medicine*.

[20] Khalatbari M. R., Moharamzad Y. (2014). Brown tumor of the spine in patients with primary hyperparathyroidism. *Spine*.

[21] Solmaz B., Tatarlı N., Günver F., Emre T. (2017). A thoracic vertebral Brown tumor presenting with paraparesis in a patient with end-stage renal disease. *British Journal of Neurosurgery*.

[22] Hong W. S., Sung M. S., Chun K.-A. (2011). Emphasis on the mr imaging findings of Brown tumor: a report of five cases. *Skeletal Radiology*.

[23] Vaishya R., Amit K., Singh H. (2019). Multiple “brown tumors” masquerading as metastatic bone disease. *World Neurosurgery*.

[24] Fargen K. M., Christine S., Lin J. A. (2013). Vertebral Brown tumors causing neurologic compromise. *World Neurosurgery*.

[25] Salamone D., Muresan S., Muresan M., Radu N. (2016). Multilevel Brown tumors of the spine in a patient with severe secondary hyperparathyroidism A case report and review of the literature. *Annali Italiani Di Chirurgia*.

[26] Sonmez E., Tezcaner T., Coven I., Terzi A. (2015). Brown tumor of the thoracic spine: first manifestation of primary hyperparathyroidism. *Journal of Korean Neurosurgical Society*.

[27] Knowles N. G., Smith D. L., Outwater E. K. (2008). MRI diagnosis of Brown tumor based on magnetic susceptibility. *Journal of Magnetic Resonance Imaging*.

[28] Khan A. A., Hanley D. A., Rizzoli R. (2017). Primary hyperparathyroidism: review and recommendations on evaluation, diagnosis, and management. A Canadian and international consensus. *Osteoporosis International*.

[29] Reséndiz-Colosia J. A., Rodríguez-Cuevas S. A., Flores-Díaz R. (2008). Evolution of maxillofacial Brown tumors after parathyroidectomy in primary hyperparathyroidism. *Head & Neck*.

[30] Yang Q., Li J., Yang Z. (2015). Skeletal lesions in primary hyperparathyroidism. *The American Journal of the Medical Sciences*.

[31] Glushko T., Banjar S. S. A., Nahal A., Colmegna I. (2015). Brown tumor of the pelvis. *Cleveland Clinic Journal of Medicine*.

[32] Gomez C. K., Schiffman S. R., Bhatt A. A. (2018). Radiological review of skull lesions. *Insights Into Imaging*.

[33] Popovik-Monevska D., Bozovik-Dvojakovska S., Popovski V., Benedetti A., Grchev A., Koneski F. (2018). Brown tumour in the mandible and skull osteosclerosis associated with primary hyperparathyroidism—a case report. *Open Access Macedonian Journal of Medical Sciences*.

[34] Bandeira L., Bilezikian J. (2016). Primary hyperparathyroidism. *F1000Research*.

[35] Marchiori D. M. (2014). Chapter 16—skull patterns. *Clinical Imaging*.

[36] Kanis J. A., Black D., Cooper C. (2002). A new approach to the development of assessment guidelines for osteoporosis. *Osteoporosis International*.

[37] Khosla S., Melton L. J. (2007). Osteopenia. *New England Journal of Medicine*.

[38] Griffith J. F., Genant H. K. (2013). Chapter 64—imaging of osteoporosis. *Osteoporosis*.

[39] Minisola S., Gianotti L., Bhadada S., Silverberg S. J. (2018). Classical complications of primary hyperparathyroidism. *Best Practice & Research Clinical Endocrinology & Metabolism*.

[40] Silva B. C., Broy S. B., Boutroy S., Schousboe J. T., Shepherd J. A., Leslie W. D. (2015). Fracture risk prediction by non-BMD DXA measures: the 2015 ISCD official positions Part 2: trabecular bone score. *Journal of Clinical Densitometry*.

[41] Boutroy S., Bouxsein M. L., Munoz F., Delmas P. D. (2005). In VivoAssessment of trabecular bone microarchitecture by high-resolution peripheral quantitative computed tomography. *The Journal of Clinical Endocrinology & Metabolism*.

[42] Parfitt A. M. (1986). Accelerated cortical bone loss: primary and secondary hyperparathyroidism. *Current Concepts of Bone Fragility*.

[43] Rubin M. R., Bilezikian J. P., McMahon D. J. (2008). The natural history of primary hyperparathyroidism with or without parathyroid surgery after 15 years. *The Journal of Clinical Endocrinology & Metabolism*.

[44] Silva B. C., Boutroy S., Zhang C. (2013). Trabecular bone score (TBS)-A novel method to evaluate bone microarchitectural texture in patients with primary hyperparathyroidism. *The Journal of Clinical Endocrinology & Metabolism*.

[45] Warzecha M., Czerwiński E., Amarowicz J., Berwecka M. (2018). Trabecular bone score (TBS) in clinical practice—rewiev. *Ortopedia Traumatologia Rehabilitacja*.

[46] Cipriani C., Abraham A., Silva B. C. (2017). Skeletal changes after restoration of the euparathyroid state in patients with hypoparathyroidism and primary hyperparathyroidism. *Endocrine*.

[47] Muschitz C., Kocijan R., Haschka J. (2015). TBS reflects trabecular microarchitecture in premenopausal women and men with idiopathic osteoporosis and low-traumatic fractures. *Bone*.

[48] Khosla S., Melton L. J., Wermers R. A., Crowson C. S., O’Fallon W. M., Riggs B. L. (1999). Primary hyperparathyroidism and the risk of fracture: a population-based study. *Journal of Bone and Mineral Research*.

[49] Vignali E., Viccica G., Diacinti D. (2009). Morphometric vertebral fractures in postmenopausal women with primary hyperparathyroidism. *The Journal of Clinical Endocrinology & Metabolism*.

[50] Walker M. D., Bilezikian J. P. (2018). Primary hyperparathyroidism. *Current Opinion in Rheumatology*.

[51] Silverberg S. J., Locker F. G., Bilezikian J. P. (1996). Vertebral osteopenia: a new indication for surgery in primary hyperparathyroidism. *Journal of Clinical Endocrinology & Metabolism*.

[52] Cusano N. E., Rubin M. R., Bilezikian J. P. (2018). Skeletal microstructure and estimated bone strength improve following parathyroidectomy in primary hyperparathyroidism. *The Journal of Clinical Endocrinology & Metabolism*.

